# The Diagnosis of the Internal Secretory Diseases*Continued from September issue.

**Published:** 1916-10

**Authors:** Henry R. Harrower

**Affiliations:** M. D., F. R. S. M. (Lond.), Los Angeles, California


### TEXAS MEDICAL JOURNAL

**Dr. F. E. Daniel, Founder. Established July, 1885**

MRS. F. E. DANIEL, - Publisher and Managing Editor

Published Monthly.—Subscription, $1.00 a Year.

VOL. XXXII AUSTIN, OCTOBER, 1916 No. 3

**The publisher is not responsible for views of contributors**

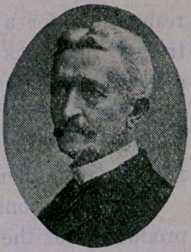

*"All of good the past hath had,*
*Remains to make our own time glad."*—WHITTIER.

### Original Articles.

### The Diagnosis of the Internal Secretory Diseases.*

BY HENRY R. HARROWER, M. D., F. R. S. M. (LOND.), LOS ANGELES, CALIFORNIA.

### ADRENAL INSUFFICIENCY.

Since the adrenals are so susceptible to so many outside influences it is likely that they would be easily "worn out," and, as a matter of fact, functional hypoadrenia is quite common. From a practical standpoint, this is an extremely important symptom complex. It is quite some years since Sajous began to emphasize the importance of this condition, and while his opinions were scouted and some of his ideas declared visionary it must be admitted that our present knowledge of this subject is very much in harmony with the following quotation from Sajous' monumental work: "Functional hypoadrenia is the symptom complex of deficient activity of the adrenals due to inadequate development, exhaustion by fatigue, senile degeneration, or any other factor which, without provoking organic lesions in the organs or their nerve paths, is capable of reducing their secretory activity. Asthenia, sensitiveness to cold and cold extremities, hypo-tension, weak cardiac action and pulse, anorexia, anemia, slow metabolism, constipation and psychasthenia are the main symptoms of this condition."

Hypoadrenia is a complication of all the serious acute infectious fevers, since the adrenals are so intimately connected with the "driving" of the body and are so susceptible to toxemia

*Continued from September issue.

that the ultimate reduction of the accustomed adrenal stimuli is responsible for a slowing down of many of the sympathetic-controlled functions of the organism. Too often this sympathetic asthenia is the actual cause of death from diseases of this character.

There are three forms of hypoadrenia which differ sufficiently from one another to be discussed separately:

(1) Functional hypoadrenia—a temporary deficiency in the production of the chromaffin hormone shown most frequently by a tardy response of the circulatory system to its accustomed stimuli and the development of a condition of circulatory inefficiency, the so-called *"hyposphyxia"* of Martinet. This is a condition of circulatory semi-asphyxia with venous stasis, inefficient arteriolar circulation with cold extremities and occasional slight blueness (often a mottled appearance) of the skin on different parts of the body, especially the exposed parts. In such individuals the blood pressure is usually very low, although it has been shown that extreme degrees of tension may cause a functional insufficiency of the adrenals by localized hemorrhage into the glands.

Urticaria and other severe vasomotor skin symptoms are among the well marked findings in persistent hyposphyxia, while lesser degrees may cause flushings and sensations of passing distress localized in various areas of the skin. The adrenal origin of some forms of urticaria is seemingly confirmed by the occasional "miraculous" disappearance of large and most uncomfortable wheals following a single hypodermic injection of from 5 to 10 minims of adrenalin solution.

With this we expect to find varying degrees of asthenia or adynamia. Exhaustion with slight cause is usual. According to Tom Williams, mental exertion, even the simplest, often causes so much weariness and exhaustion as to be prohibitive. Mental elasticity is lost and there is both mental and physical depression with the fear that the individual cannot now accomplish their accustomed good mental work; and the story that they "have lost their nerve." With this one frequently notes a fearfulness of making wrong decisions and a vacillating and indecisive frame of mind.

(2) Progressive hypoadrenia. Here we expect more than the mere functional derangement. This is really another name for the disease we have been taught was first named by Addison in 1855, which, like all organic diseases, may be seen in differing forms and stages. The main symptom is the aggravated asthenia with marked myasthenia. In well advanced cases there is a localized bronzing of the skin and mucous membranes due to the deposition of a dark pigment of undecided origin. Extreme cardiovascular debility is the rule and the blood pressure may be as low as 30 to 50 mm. Hg. Varying gastro-intestinal disturbances are usual. Fortunately this disease is rare and unfortunately its outcome is hopeless, though temporary relief has followed adrenal medication.

Lawrence in his recent consideration of "Some Aspects of Hypotension" connects hypofunction of the adrenal glands with weakness and apathy, marked fatigability and a tendency toward vertigo. These are merely variations in degree of the classical symptoms first reported by Addison.

(3) Terminal hypoadrenia. This is the extreme functional adrenal insufficiency which has already been briefly mentioned. This occurs in the final stages of fatal infectious diseases. For instance, the principal clinical manifestations of Asiatic cholera (the algid stage) are adrenal in origin and, remarkably enough, have been promptly and successfully controlled by heroic doses of adrenalin, for in such cases the tolerance of this drug is apparently greatly increased and as much as an ounce of the commercial 1:1000 solution well diluted with saline solution has been given intravenously during a single day with splendid results (Naamé).

Shock, collapse, cardiac failure and distressing asthenia are terminal findings in this class of cases. Distressing meteorism is present and is presumably due to functional intestinal paresis, which, by the way, can be experimentally produced by fright or toxemia and the resulting acute hypoadrenia. With these dread symptoms there is often found a noticeable reduction in the reaction of the organisms to urgently needed medication, for with the adrenal activities suspended the responsiveness of the body to stimuli of this character is practically nil.

The ominous sign of a suddenly reduced temperature is often seen and is due to the same cause. In such cases one can invariably produce Sergent's *"ligne blanche surrénale,"* a dermographic sign which consists of a white line upon the skin which follows pencilling the abdomen with the finger nail, and sometimes lasts for two or three minutes. This valuable clinical sign is said to be pathognomonic of acute hypoadrenia and is very easily elicited.

In cases of the character just considered, despite their severity, the therapeutic test is often both encouraging and confirmatory, for the response to hypodermic or intravenous injections of adrenalin solution, and, in many cases, the early administration of this remedy by mouth, is many times nothing short of marvelous. At times I feel that this phase of adrenal medication deserves to be classed with thyroid in myxedema and quinine in malaria. At least it is worth recommending both as a prophylactic of such ultimate results and as a last resort in their treatment.

### NEURASTHENIA AS AN ADRENAL SYNDROME.

This chapter should not be closed without a short consideration of the relation of adrenal dysfunction to neurasthenia. Minor functional hypoadrenia is more common than some have appreciated, and the fact that there is a psychic origin as well as the other physiologic causes already considered, allies it to the fashionable "neurasthenia" of today. In fact some have stated that what is improperly called "neurasthenia" is not a disease *per se,* but really a symptom complex of ductless glandular origin and that the adrenals are probably the most important factors in its causation. Campbell Smith, Osborne, Williams and others, including the writer, have directed the attention of the profession to the importance of the adrenal origin of neurasthenia (though a pluriglandular dyscrasia is practically always discoverable) but so far this is not understood as well as its frequency and importance warrant.

The subject is too large to receive exhaustive consideration here, but a few quotations from recent literature will firmly establish the importance of this angle from which to study this common and annoying symptom complex.

Quoting first from the *Journal A. M. A.* (December 18, 1915, page 2166): "The typical neurotic generally has, if not always, disturbance of the thyroid gland. The typical neurasthenic probably generally has disturbance of the suprarenal glands on the side of insufficiency. The blood pressure in these neurasthenic patients is almost always low for the individuals, and their circulation is poor. * * * A vasomotor paralysis, often present, allows chillings, flushings, cold or burning hands and feet, drowsiness when the patient is up, wakefulness on lying down and hence insomnia. There may be more or less tingling or numbness of the extremities."

Again Kinnier Wilson in his monograph on "The Clinical Importance of the Sympathetic Nervous System" makes the following pertinent remarks: "Many of the common symptoms of neurasthenia and hysteria are patently of sympathetic origin. Who of us has not seen the typical irregular blotches appear on the skin of the neck and face as the neurasthenic subject 'works himself up into a state'? The clammy hand, flushed or pallid features, dilated pupils, the innumerable parentheses, the unwonted sensations in head or body, are surely of sympathetic parentage. In not a few cases of neurasthenia symptoms of this class are the chief or only manifestations of the disease. Here, then, is a condition of defective sympatheticotonus; may it not have much to do with impairment of function of the chromophil system? * * * There does not appear to me any tenable distinction between the asthenia of Addison's disease and the asthenia of neurasthenia. Cases of the former are not infrequently diagnosed as ordinary neurasthenia at first. It is difficult to avoid the conclusion that defect of glandular function is responsible for much of the clinical picture of neurasthenia."

Later this same author makes the following apothegm: "Sympathetic tone is dependent on adrenal support, and until the glandular equilibrium is once more attained sympathetic symptoms are likely to occur."

ADRENIN SENSIBILITY.

As the art of clinical diagnosis is perfected we are having brought to our attention numerous "tests" of function and several of these are dependent upon a condition of hormone balance involving the adrenals. This has been called "adrenin sensibility" and the best known test based on this is that of Loewi, which consists of instilling a few drops of adrenalin solution (1:1000) into the conjunctival sac. Without going into a rather complicated subject—the study of the hormone balance is not yet very simple—marked dilatation of the pupil occurs in thirty to sixty minutes in pancreatic diabetes, and this test has been used to confirm suspicions as to the waning internal secretory powers of the pancreas of which more will be said in a later chapter.

This same test is of value in Graves' disease and a dilatation following conjunctival instillations of adrenalin solution has also been proposed as a means of determining thyroid hyperfunction. We cannot take space to explain the *raison d'être* of these tests but recommend them as confirmatory measures only.

Schultz has advanced an almost identical test for incipient dementia praecox and asserts that marked mydriasis will follow in about ten minutes after the instillation as above and that the pupillary dilatation will last as long as half an hour. Parenthetically it may be well to remark that Dunlop Robertson believes that the catatonic type of dementia praecox is due to hyperadrenia.

715 Baker Detwiler Building.

---

The title of the next article in this series will be "The Diagnosis of Disorders of the Pituitary Body." Attention is again directed to the opportunity afforded to readers of TEXAS MEDICAL JOURNAL to send in questions or short case reports for consideration and answer in these columns. There is no charge for this service.—ED.

